# Effect of a Very-Low-Protein Diet Supplemented with Ketoacid Analogues on Arteriovenous Fistula Maturation and Endothelial Function: A Prospective Observational Study

**DOI:** 10.3390/nu18111777

**Published:** 2026-05-31

**Authors:** Silvia Barbarini, Paolo Protopapa, Giulia Fontò, Paolo Ria, Alessandra Pesino, Anna Zito, Stefano Scardia, Stefania Maria Pia Doronzo, Marcello Napoli, Antonio De Pascalis

**Affiliations:** 1 Nephrology Department, Vito Fazzi Hospital, ASL Lecce, 73100 Lecce, Italyria.paolo@gmail.com (P.R.); ale.pesino@gmail.com (A.P.); anna.zito.84@gmail.com (A.Z.);; 2 Clinical Nutrition Department, Vito Fazzi Hospital, ASL Lecce, 73100 Lecce, Italy; 3 INSPIRE Lab, Department of Biological and Environmental Sciences and Technologies, University of Salento, 73100 Lecce, Italy

**Keywords:** chronic kidney disease, very-low-protein diet, ketoacid analogues, arteriovenous fistula, endothelial function, uremic toxins

## Abstract

Background: Arteriovenous fistula (AVF) maturation is a critical determinant for successful hemodialysis in patients with end-stage renal disease (ESRD). Endothelial dysfunction and arterial stiffness, which are highly prevalent in advanced chronic kidney disease (CKD), frequently impair AVF maturation. Emerging evidence suggests that a very-low-protein diet (VLPD) supplemented with ketoacid analogues (KA) mitigates nitrogenous waste accumulation and positively influences vascular health by reducing inflammation and improving endothelial function. This prospective observational study evaluates the effect of VLPD+KA on AVF maturation, endothelial function, inflammatory markers, nutritional status, and the timing of dialysis initiation. We enrolled 20 patients with advanced CKD (stage V) scheduled for AVF creation. Participants adhered to a strict VLPD protocol (0.3–0.4 g/kg/day) with KA supplementation (1 tablet/5 kg/day). We assessed biochemical parameters, inflammatory markers (CRP, ESR), uremic toxins (indoxyl sulfate [IS], p-cresyl sulfate [PCS]), endothelial function via flow-mediated dilation (FMD), and vascular imaging metrics. AVF maturation, central venous catheter (CVC) requirement, dialysis initiation, and nutritional parameters were monitored over a three-month follow-up period. Statistical analyses included paired t-tests or Wilcoxon signed-rank tests for within-group comparisons, Fisher’s exact test for categorical variables, and Kaplan–Meier analysis for time-to-event endpoints, performed using SPSS version 27.0. Results: The intervention led to significant improvements in endothelial function (FMD +1.7%, *p* < 0.01), substantial reductions in the uremic toxins IS (−38%, *p* < 0.001) and PCS (−43%, *p* < 0.001), and a marked decrease in CRP levels (from 3.2 to 1.1 mg/L, *p* < 0.01). Nutritional status, assessed by BMI, BIA-derived phase angle, and handgrip strength, remained stable throughout the intervention period, confirming the metabolic safety of the VLPD+KA regimen. Notably, AVF maturation was achieved in 95% of patients, with zero CVC dependency among those who initiated dialysis. Conclusions: These findings strongly support the hypothesis that VLPD+KA therapy enhances vascular integrity, reduces uremic endotheliotoxicity, and facilitates successful AVF maturation. This nutritional intervention warrants further investigation in larger, randomized controlled trials as a standard pre-dialysis care strategy.

## 1. Introduction

Chronic kidney disease (CKD) represents a rapidly growing global public health challenge, affecting nearly 10% of the world’s population. Its progression to end-stage renal disease (ESRD) imposes a significant burden on healthcare systems, primarily due to the necessity for renal replacement therapy (RRT).

Individuals undergoing dialysis face a substantially elevated risk of cardiovascular disease (CVD) compared to the general population, a condition that carries considerable morbidity and mortality burdens. A 2022 analysis conducted by Bello and colleagues revealed striking disparities in life expectancy: among adults between 40 and 44 years of age, dialysis patients live approximately 20 to 25 years less than their counterparts in the general population. Even among older individuals aged 80 to 84, those receiving dialysis still face a reduction in life expectancy of 2 to 5 years relative to peers of the same age. Notably, cardiovascular risk tends to increase as the estimated glomerular filtration rate (eGFR) declines and as proteinuria worsens.

The importance of combining both pharmacological and non-pharmacological strategies to delay the progression of CKD toward end-stage renal disease has been reinforced by the most recent KDIGO 2024 guidelines. Among first-line pharmacological options, the recommendations include sodium–glucose cotransporter-2 inhibitors, renin–angiotensin system inhibitors, and lipid-lowering therapy. Beyond medications, lifestyle modifications are equally critical: these encompass a balanced diet, regular physical exercise, complete avoidance of tobacco, and effective body weight control. For CKD patients specifically, at least 150 min per week of moderate-intensity physical activity is advised, with adjustments made according to the individual’s age and existing comorbidities. From a dietary standpoint, an approach resembling the DASH (Dietary Approaches to Stop Hypertension) diet is recommended for CKD patients. This consists primarily of plant-based foods with limited intake of animal-derived products and sodium, while minimizing ultra-processed items such as sugary drinks, frozen ready meals, and fast food, all of which offer poor nutritional value. The rationale behind this plant-based strategy lies in its ability to foster a healthier gut microbiome by promoting the growth of commensal bacteria, thereby reducing systemic inflammation and limiting the generation of uremic toxins. Furthermore, plant-based dietary patterns—whether Mediterranean, vegetarian, or vegan—that emphasize unprocessed protein sources have been shown to attenuate eGFR decline, lower proteinuria, and mitigate metabolic acidosis.

As renal function declines, patients develop a complex pro-inflammatory and pro-oxidative state known as uremia, which induces profound alterations in vascular structure and function. This mechanism is connected with proteolytic metabolism in the dysbiotic gut. Three of the most well-studied and damaging toxins are Indoxyl Sulfate (IS), p-Cresyl Sulfate (PCS), and Trimethylamine-N-Oxide (TMAO).

Among the most clinically relevant protein-bound uremic toxins are indoxyl sulfate (IS), p-cresyl sulfate (PCS), and trimethylamine-N-oxide (TMAO). IS is derived from the bacterial metabolism of tryptophan and promotes endothelial dysfunction and CKD progression. PCS, generated from tyrosine and phenylalanine, is a powerful predictor of all-cause mortality and contributes to systemic inflammation and protein-energy wasting. TMAO, produced from dietary choline and carnitine, is a major driver of atherosclerosis and cardiovascular risk. Together, these toxins overwhelm the failing kidneys’ clearance capacity and directly fuel cardiovascular disease, fibrosis, and malnutrition in a self-perpetuating feedback loop.

This “uremic vasculopathy” is characterized by endothelial dysfunction, accelerated arterial stiffness, vascular calcification, and maladaptive remodeling of blood vessels.

In this context, the creation of a functional and durable vascular access is paramount for effective hemodialysis. The arteriovenous fistula (AVF), surgically created by anastomosing an artery and a vein, remains the access of choice due to its superior long-term patency and significantly lower rates of infectious and thrombotic complications compared to central venous catheters (CVCs) and prosthetic grafts. However, the success of an AVF depends on a complex biological process known as maturation, which requires adaptive vascular remodeling, endothelium-mediated vasodilation, and a substantial increase in blood flow. Unfortunately, the rate of AVF maturation failure remains unacceptably high. Recent contemporary cohorts and systematic reviews report failure rates ranging from 25% to 60%, with primary non-function rates persisting despite advances in surgical techniques and preoperative mapping. This high failure rate leads to multiple surgical revisions, prolonged CVC use, and increased patient morbidity.

The pathophysiology of AVF maturation failure is multifactorial, yet endothelial dysfunction plays a central and initiating role. The uremic environment impairs the endothelium’s ability to produce nitric oxide (NO), a potent vasodilator and inhibitor of platelet aggregation and smooth muscle cell proliferation. IS and PCS exacerbate this state by downregulating endothelial NO synthase (eNOS), inducing oxidative stress, and upregulating pro-inflammatory cytokines and adhesion molecules such as MCP-1 and IL-8. This cascade promotes neointimal hyperplasia—the primary pathological process underlying venous stenosis and AVF failure—and represents a compelling therapeutic target for nutritional intervention.

Nutritional intervention is a cornerstone of the conservative management of advanced CKD. Low-protein diets (LPD) and very-low-protein diets (VLPD) aim to reduce the production of nitrogenous waste products, mitigating uremic symptoms and potentially delaying the need for dialysis. However, severe protein restriction raises valid concerns regarding the risk of protein-energy wasting (PEW). To circumvent this issue, supplementation with ketoacid analogues (KA) of essential amino acids offers a strategic metabolic solution. These nitrogen-free precursors can be transaminated into their corresponding amino acids, recycling endogenous nitrogen for protein synthesis and thus maintaining a neutral nitrogen balance. Beyond their nutritional benefits, recent studies suggest that VLPD+KA therapy exerts pleiotropic effects, including the modulation of the gut microbiota. By reducing the protein substrate available for fermentation, VLPD+KA can alter the gut microbial composition, reducing the generation of IS and PCS and subsequently improving endothelial function. This prospective study was designed to investigate the hypothesis that a targeted nutritional intervention with VLPD+KA can improve AVF maturation outcomes, reduce CVC dependency, and enhance vascular and inflammatory parameters in ESRD patients awaiting hemodialysis.

## 2. Materials and Methods

### 2.1. Study Design and Setting

This was a single-center, prospective, observational cohort study conducted at the Department of Nephrology of a tertiary care hospital (Vito Fazzi Hospital, Lecce, Italy). The study period extended from January to September 2024. All study protocols were rigorously reviewed and approved by the Institutional Review Board (Ethics Committee) of Vito Fazzi Hospital, Lecce, Italy (protocol code 2024-01, approved 27 January 2024), and conducted in full accordance with the principles of the Declaration of Helsinki. Written informed consent was obtained from all participants prior to enrolment.

### 2.2. Patient Population

Eligible participants were adults aged 18 years or older with stage V CKD (estimated glomerular filtration rate [eGFR] < 15 mL/min/1.73 m^2^) who were referred for primary AVF creation. Exclusion criteria included a history of KA intolerance, active malignancy, liver cirrhosis, recent acute infections, prior prosthetic access, or advanced cardiovascular disease (e.g., recent myocardial infarction, decompensated heart failure). Patients already undergoing dialysis or those previously adhering to any form of protein-restricted diet were also excluded to prevent confounding baseline variables. All AVFs were created at the distal forearm as radiocephalic fistulas at the wrist, which represents the preferred first-choice access in accordance with the KDOQI Vascular Access Guidelines [1]. Pre-operative vascular mapping by Doppler ultrasound confirmed adequate vessel calibre in all patients (pre-operative cephalic vein diameter: 2.8 ± 0.4 mm; radial artery diameter: 2.2 ± 0.3 mm).

### 2.3. Nutritional Intervention

Participants were instructed to follow a VLPD providing 0.3 to 0.4 g of protein per kilogram of ideal body weight per day. Dietary protein consisted predominantly of high biological value sources, principally egg white and dairy products, in accordance with standard VLPD protocols for advanced CKD. The diet was supplemented with KA (Ketosteril), dosed at one tablet per 5 kg of body weight per day, divided into three daily doses administered with meals. Dietary adherence was ensured through a structured, multi-modal monitoring approach: (i) bi-weekly individualized dietary counseling sessions with expert renal dietitians, including personalized meal planning and ongoing nutritional education; (ii) objective compliance monitoring via 24-h urinary urea nitrogen (UUN) measurements, which served as a reliable biochemical marker of actual protein intake; and (iii) subjective dietary assessment using standardized 3-day weighed dietary records, completed by patients at regular intervals throughout the follow-up period. This dual biochemical and self-reported monitoring strategy allowed cross-validation of adherence and timely identification of any deviations from the prescribed regimen. Total energy intake was strictly maintained at 30–35 kcal/kg/day to prevent catabolism and protein-energy wasting (PEW).

### 2.4. Laboratory and Imaging Assessments

All laboratory and imaging assessments were performed at four pre-specified time points: baseline (prior to AVF creation) and at 1, 2, and 3 months post-operatively. Routine blood tests included a comprehensive metabolic panel, calcium–phosphate balance, bicarbonate, serum albumin, lipid profile, C-reactive protein (CRP), erythrocyte sedimentation rate (ESR), and intact parathyroid hormone (iPTH). The eGFR was calculated using the Modification of Diet in Renal Disease (MDRD) equation.

The uremic toxins IS and PCS were quantified using high-performance liquid chromatography (HPLC) coupled with fluorescence detection. Briefly, serum samples underwent deproteinization using methanol to remove interfering proteins. The analysis utilized a mobile phase composed of phosphate buffer and acetonitrile to achieve optimal separation. Fluorescence detection was set at specific excitation/emission wavelengths to ensure high sensitivity and specificity for IS and PCS quantification.

Endothelial function was assessed by measuring the flow-mediated dilation (FMD) of the brachial artery using high-resolution Doppler ultrasound, adhering to established international guidelines [2]. A linear array transducer (7.5–12 MHz) was used to image the brachial artery. After obtaining a baseline image, a pneumatic cuff was placed on the forearm and inflated to 50 mmHg above the patient’s systolic blood pressure for 5 min to induce ischemia. Post-deflation images were recorded continuously, and the peak arterial diameter was measured between 60 and 90 s after cuff release. FMD was calculated as the percentage change from baseline. Vascular imaging also included the measurement of intima-media thickness, vessel diameter (pre- and post-tourniquet), resistance index (RI), and AVF flow volume by Color Doppler. To minimize inter-observer variability, all imaging was conducted by a single, highly experienced vascular sonographer using a standardized protocol.

### 2.5. Nutritional Assessment

Nutritional assessments were performed at the same four time points as laboratory evaluations: baseline and at 1, 2, and 3 months. Nutritional status was comprehensively evaluated using Body Mass Index (BMI), bioelectrical impedance analysis (BIA)-derived phase angle, and handgrip strength measured with a calibrated dynamometer. These assessments followed KDOQI and KDIGO guidelines and were interpreted against sex- and age-specific normative reference values. Additionally, the appendicular skeletal muscle mass (ASM) derived from BIA was used to calculate the Skeletal Muscle Mass Index (SMMI = ASM/height^2^, kg/m^2^), a validated measure of muscle quantity recommended by EWGSOP2 for the diagnosis of sarcopenia. Sex-specific SMMI cut-offs were applied: <7.0 kg/m^2^ in men and <5.5 kg/m^2^ in women. Handgrip strength data were additionally reported stratified by sex, using EWGSOP2 cut-offs of <27 kg for men and <16 kg for women. BIA-derived phase angle values below the 5th percentile for the corresponding sex and age group were considered indicative of nutritional risk; in our cohort (mean age 62 ± 11 years), values ≤ 4.0° were used as the lower threshold of adequacy. Handgrip strength was interpreted using sex- and age-specific normative cut-offs, with low muscle strength defined as <27 kg in men and <16 kg in women, in accordance with the European Working Group on Sarcopenia in Older People 2 (EWGSOP2) criteria.

### 2.6. Endpoints and Statistical Analysis

The primary endpoint was the time from AVF creation to dialysis initiation without the need for a CVC. Secondary endpoints included AVF maturation rates at 1, 2, and 3 months; the need for endovascular interventions; reduction in IS and PCS levels; improvements in FMD and CRP; and changes in nutritional status.

Data were analyzed using SPSS version 27.0. Continuous variables were presented as mean ± standard deviation (SD) or median (interquartile range [IQR]) as appropriate based on data distribution. Categorical variables were expressed as counts and percentages. Paired t-tests or Wilcoxon signed-rank tests were utilized for within-group comparisons from baseline to 3 months. Kaplan–Meier curves were generated to analyze CVC-free survival. A *p*-value of < 0.05 was considered statistically significant.

## 3. Results

### 3.1. Baseline Characteristics

A total of 20 patients (12 male and 8 female) with a mean age of 62 ± 11 years were enrolled in the study. The most common etiologies of CKD were diabetic nephropathy (40%), hypertensive nephrosclerosis (30%), and chronic glomerulonephritis (15%). At baseline, the mean eGFR was 10.2 ± 2.3 mL/min/1.73 m^2^, and all participants were dialysis-naive. Nutritional status was well-preserved at baseline, with a mean BMI of 24.7 ± 2.6 kg/m^2^, a handgrip strength of 28.5 ± 4.8 kg, and a BIA phase angle of 4.5 ± 0.7 degrees. Serum albumin was within normal limits (3.9 ± 0.3 g/dL), indicating an absence of overt protein-energy wasting prior to the intervention. Complete baseline biochemical data, including renal function parameters, mineral metabolism, haematological indices, and lipid profile, alongside 3-month follow-up values, are reported in Table 1. Dietary compliance data and KA supplementation details are summarized in Table 2. No patient withdrew from the study or was lost to follow-up during the 3-month observation period; all 20 enrolled patients completed the full assessment protocol and are included in the final analysis.

### 3.2. AVF Maturation and Dialysis Initiation

All 20 AVFs were created as distal radiocephalic fistulas at the wrist. Clinical and ultrasonographic AVF maturation was successfully achieved in 19 of the 20 patients (95%) within three months of surgical creation. Maturation rates at sequential monthly assessments were 60% (12/20) at 1 month, 85% (17/20) at 2 months, and 95% (19/20) at 3 months. AVF blood flow volume, measured by Color Doppler, increased progressively from 420 ± 95 mL/min at 1 month to 558 ± 88 mL/min at 2 months and 648 ± 102 mL/min at 3 months, meeting the KDOQI maturation threshold of ≥600 mL/min. Cephalic vein diameter increased from 4.2 ± 0.6 mm at 1 month to 5.4 ± 0.7 mm at 2 months and 6.3 ± 0.8 mm at 3 months, exceeding the KDOQI criterion of ≥6 mm. These longitudinal AVF maturation data are summarized in Table 3. Only one patient required a salvage balloon angioplasty due to juxta-anastomotic venous stenosis, which subsequently matured. Remarkably, none of the patients required a central venous catheter for initial dialysis access during the study period. Dialysis was initiated in 4 patients (20%) by the end of the 3-month follow-up, and all 4 successfully initiated hemodialysis using their mature AVF. The median time to dialysis initiation was 81 days (IQR: 71–94 days). Kaplan–Meier survival analysis for CVC-free dialysis initiation demonstrated a highly favorable profile, with 100% of patients remaining CVC-free at day 60 and 80% at day 90. All biochemical assessments—including IS and PCS measurements—were completed for all 4 patients who initiated dialysis prior to or at the time of dialysis start; their data are fully included in the 3-month endpoint analysis and contribute to the within-group comparisons reported in Table 4.

### 3.3. Changes in Vascular, Inflammatory, and Nutritional Parameters

The changes in key biomarkers from baseline to the 3-month follow-up are summarized in Table 1. Statistically significant improvements were observed in endothelial function, uremic toxin clearance, and systemic markers of inflammation, while nutritional status remained stable despite the severe protein restriction. Sarcopenia was formally evaluated in all patients according to the EWGSOP2 diagnostic criteria, combining assessment of muscle strength (handgrip) and muscle mass/quality (BIA-derived phase angle). At baseline, none of the 20 enrolled patients met the EWGSOP2 criteria for sarcopenia: mean handgrip strength was 28.5 ± 4.8 kg—above the sex-specific cut-off thresholds (<27 kg in men, <16 kg in women) in all patients—and mean BIA-derived phase angle was 4.5 ± 0.7°, consistent with preserved muscle mass for the cohort’s age and sex profile. At 3-month follow-up, both parameters remained stable and within normal limits (handgrip: 29.1 ± 4.8 kg, *p* = n.s.; phase angle: 4.7 ± 0.7°, *p* = 0.04), confirming the complete absence of sarcopenia onset or progression during the intervention period. This finding constitutes an important safety outcome of the VLPD+KA regimen: KA supplementation, by providing essential nitrogen-free amino acid analogues, effectively prevented the skeletal muscle catabolism that could otherwise be expected with such a severely restricted protein intake in a CKD population at high nutritional risk, distinguishing this approach from unsupplemented very-low-protein diets, which carry a recognized risk of protein-energy wasting and sarcopenia progression. Sex-stratified analysis of handgrip strength showed values consistently above EWGSOP2 thresholds in both groups throughout the study: in males (n = 12), mean handgrip was 33.2 ± 3.8 kg at baseline and 33.9 ± 3.7 kg at 3 months (*p* = n.s.); in females (n = 8), mean handgrip was 21.4 ± 3.1 kg at baseline and 21.8 ± 3.0 kg at 3 months (*p* = n.s.). Mean SMMI was 8.2 ± 0.9 kg/m^2^ in males and 6.1 ± 0.7 kg/m^2^ in females at baseline, remaining stable at 3 months (males: 8.3 ± 0.8 kg/m^2^; females: 6.2 ± 0.6 kg/m^2^; both *p* = n.s.), above the respective EWGSOP2 cut-offs of 7.0 and 5.5 kg/m^2^. These data confirm the absence of low muscle mass or sarcopenia in any patient throughout the intervention. A pre-specified sensitivity analysis was performed, excluding the 4 patients who initiated dialysis during the follow-up period, to assess whether dialysis-related clearance could have confounded the observed reductions in IS and PCS. It is well established that IS and PCS are tightly protein-bound uremic toxins that are poorly removed by conventional hemodialysis, owing to their high affinity for albumin binding sites and their large volume of distribution. Consistent with this biological property, the sensitivity analysis (n = 16) yielded reductions in IS of −34.3% (from 31.4 ± 8.2 to 20.6 ± 6.0 μmol/L, *p* < 0.001) and in PCS of −40.0% (from 22.7 ± 5.5 to 13.6 ± 4.5 μmol/L, *p* < 0.001), both remaining highly significant and quantitatively close to the primary analysis (IS: −38.0%; PCS: −43.0%). The difference between the two analyses was less than 4 percentage points for both toxins, confirming that the contribution of dialysis-related clearance to the observed reductions was negligible. On the basis of these findings, the decision was made to retain all 20 patients in the primary analysis, as the exclusion of the dialysis subgroup did not materially alter the conclusions of the study.

## 4. Discussion

### 4.1. Interpretation of Findings and Mechanistic Insights

This study provides compelling evidence that a targeted nutritional intervention, combining a very-low-protein diet (VLPD) with ketoanalogue (KA) supplementation, confers significant vascular and metabolic benefits in patients with advanced chronic kidney disease (CKD) awaiting hemodialysis. The most striking clinical finding is the exceptional arteriovenous fistula (AVF) maturation rate of 95%, accompanied by a complete absence of central venous catheter (CVC) requirement for dialysis initiation. This outcome stands in sharp contrast with rates reported in the contemporary literature, where multicenter studies and recent meta-analyses document AVF maturation rates ranging from 40% to 70% in general CKD populations, with primary failure rates remaining a persistent clinical concern [1,3]. The superior result observed in our cohort suggests that the VLPD+KA regimen not only preserved vascular health but also actively improved it, creating a highly favorable hemodynamic and cellular environment for fistula remodeling. This success is likely attributable to a synergistic combination of careful patient selection, proactive nutritional optimization initiated well before AVF creation, and the direct anti-inflammatory and endothelial-protective effects of the dietary intervention. Importantly, this study was intentionally designed as a first, exploratory contribution to characterize the relationship between VLPD+KA supplementation, vascular endothelial health, and AVF maturation outcomes—a clinical dimension of this therapeutic approach that has not been previously described in the literature. A particularly meaningful implication of our findings is that the time gained by delaying dialysis initiation through nutritional therapy creates a critical window to achieve adequate AVF maturation, thereby avoiding central venous catheter (CVC) placement. This benefit is especially relevant for late-referral patients, who—in the absence of pre-dialysis nutritional optimization—would otherwise have required urgent CVC insertion at the time of dialysis initiation, with all the associated infectious, thrombotic, and hemodynamic risks.

The improvement in flow-mediated dilatation (FMD) of the brachial artery, a validated non-invasive surrogate for endothelial function and an independent predictor of cardiovascular (CV) risk, is of particular mechanistic relevance. The mean absolute increase of 1.7% is both statistically and clinically significant, indicating a restoration of endothelium-dependent vasodilatory capacity. FMD is driven by the shear-stress-induced release of nitric oxide (NO) from endothelial cells, and its improvement directly reflects an increase in NO bioavailability [2]. A healthier endothelium, capable of mounting an adequate vasodilatory response, facilitates the outward remodeling and shear-stress-mediated dilation necessary for successful AVF maturation. This restoration of endothelial function is therefore likely the primary physiological driver behind the superior AVF outcomes observed in our cohort. In this context, it is worth noting that FMD has been correlated with coronary endothelial function and carotid intima-media thickness, underscoring its value as a systemic vascular health marker rather than a purely peripheral measure [4]. The present finding thus carries implications extending well beyond hemodialysis access, pointing toward a broad reduction of CV risk in this high-risk population.

The substantial and rapid reductions in serum indoxyl sulfate (IS; −38%) and p-cresyl sulfate (PCS; −43%) levels provide a compelling molecular explanation for the observed vascular improvements. These protein-bound uremic toxins, generated primarily by intestinal bacterial fermentation of dietary protein, are established endotheliotoxins: they induce oxidative stress, downregulate endothelial NO synthase (eNOS) activity, reduce NO bioavailability, promote neointimal hyperplasia, and stimulate vascular smooth muscle cell (VSMC) proliferation [5,6]. Their reduction alleviated a massive toxic burden on the vasculature. These findings align with the growing understanding of the gut-kidney axis in the pathogenesis of uremia. By drastically reducing the protein substrate available for intestinal bacterial fermentation, the VLPD limits the generation of these toxins. Furthermore, KAs may modulate the gut microbiota composition and alter nitrogen metabolism, thereby enhancing the clearance of IS and PCS and improving the ratio of saccharolytic to proteolytic fermentation [7]. CKD patients are characterized by gut dysbiosis—an imbalance in the microbial community favoring proteolytic species at the expense of saccharolytic ones—resulting in decreased short-chain fatty acid (SCFA) production and increased uremic toxin generation [8]. The VLPD+KA intervention directly addresses this dysbiosis by reducing the nitrogenous substrate that drives proteolytic fermentation, thus modulating the gut–kidney axis in a therapeutically favorable direction. It should be noted that, in the present single-arm study, formal dissociation of the respective contributions of severe protein restriction and KA supplementation to the observed IS and PCS reductions was not possible. Both components plausibly contribute through distinct but complementary mechanisms: the VLPD drastically reduces the dietary protein substrate available for intestinal bacterial fermentation—the primary biosynthetic source of IS and PCS—while KA supplementation may independently modulate gut microbiota composition and nitrogen recycling, shifting fermentation from a proteolytic toward a saccharolytic pattern. The dietary protein in our protocol consisted predominantly of high biological value (HBV) sources—principally egg white and low-fat dairy products—which, while providing essential amino acids with high nitrogen efficiency, are relatively low in aromatic precursors such as tryptophan, tyrosine, and phenylalanine, the primary dietary substrates for IS and PCS generation respectively. To compensate for the severe protein restriction while maintaining adequate energy intake (30–35 kcal/kg/day), patients followed a diet rich in complex carbohydrates (including low-protein specialty pasta, bread, and rice), extra virgin olive oil as the primary fat source, and an abundance of non-starchy vegetables and fruits—foods that are naturally devoid of nitrogenous fermentation substrates and thus contribute negligibly to uremic toxin generation. This specific dietary composition, by minimizing the supply of aromatic amino acid precursors to the gut microbiota, is directly relevant to the observed toxin reductions and represents a dimension of the intervention that extends beyond the pharmacological action of KA supplementation alone. Importantly, the protein-bound nature of IS and PCS renders them poorly amenable to removal by conventional hemodialysis: their tight binding to albumin limits free-fraction availability for diffusive or convective clearance, resulting in reduction ratios of typically less than 20–30% per dialysis session—far below the 38–43% reductions observed over three months in the present study. A sensitivity analysis excluding the 4 patients who initiated dialysis during the follow-up confirmed that the reductions in IS (−34.3%, *p* < 0.001) and PCS (−40.0%, *p* < 0.001) in the non-dialysis subgroup (n = 16) were virtually identical to those of the full cohort, providing robust evidence that the observed toxin reductions are attributable to the dietary intervention rather than to dialysis-related clearance.

The parallel decrease in systemic inflammatory markers—namely C-reactive protein (CRP) and erythrocyte sedimentation rate (ESR)—further corroborates the anti-inflammatory mechanism of action of the VLPD+KA regimen. Low-grade chronic systemic inflammation is a hallmark of CKD, involving activation of the nuclear factor kappa-B (NF-kB) pathway, increased secretion of interleukin (IL)-1beta, IL-6, and tumor necrosis factor-alpha (TNF-alpha), and consequent reduction in eNOS enzyme activity [9]. The reduction of IS and PCS likely mitigates the activation of these inflammatory pathways, potentially including the aryl hydrocarbon receptor (AhR) signaling pathway, which IS is known to activate and which has been implicated in vascular inflammation, VSMC remodeling, and accelerated atherogenesis [10]. Moreover, chronic low-grade inflammation in CKD is recognized as a key driver not only of CV disease but also of protein-energy wasting (PEW) syndrome, a comorbidity that further compromises vascular integrity and surgical outcomes [9]. By reducing the inflammatory burden, the VLPD+KA regimen may therefore simultaneously protect the endothelium, preserve nutritional status, and improve overall prognosis.

### 4.2. Comparison with Existing Literature

Our findings are consistent with and significantly expand upon previous landmark studies by Garneata et al. and Brunori et al., who documented improved metabolic profiles and a significant delay in dialysis initiation with VLPD+KA supplementation in patients with advanced CKD [11,12]. While those studies primarily focused on renal function preservation and metabolic benefits, our study is among the first to specifically evaluate the vascular implications of this therapy in the critical and time-sensitive context of hemodialysis access preparation. The nutritional and biochemical improvements previously described—including reductions in urea, phosphate, metabolic acidosis, and uremic toxin burden—are expanded in the present work to include direct, quantifiable improvements in endothelial function as assessed by FMD. This is particularly relevant because, as demonstrated by Chang et al. in a cohort of CKD stage 3b-4 patients, LPD combined with KA supplementation significantly decreased IS and PCS levels and led to a measurable increase in FMD, reflecting enhanced NO production and reduced endothelial damage [13]. Our data in a VLPD+KA setting thus extend this evidence to a more advanced CKD population with an even more clinically meaningful endpoint, namely AVF maturation of particular relevance to the present work is the study by David et al. [14], who were the first to demonstrate that VLPD supplemented with KA/EAA can improve AVF creation success rates and shorten maturation time in pre-dialysis ESRD patients. We fully acknowledge their seminal contribution and recognize that our claim of novelty requires precise qualification in light of this reference. However, a careful comparison reveals that our study substantially extends their work across multiple dimensions.

Regarding dietary protocol, David et al. prescribed a protein intake of 0.4–0.6 g/kg/day with KA supplementation—a standard low-protein diet (LPD) rather than a true VLPD—whereas our protocol imposed a significantly stricter restriction of 0.3–0.4 g/kg/day, confirmed by objective 24-h urinary urea nitrogen monitoring (UUN 2.9 ± 0.6 g/24 h at 3 months). This distinction is clinically meaningful, as a more severe reduction in dietary protein substrate directly limits the generation of gut-derived uremic toxins. Importantly, David et al. acknowledged the absence of UUN measurements as an explicit limitation of their compliance assessment, which relied retrospectively on medical records and patient self-report; our dual monitoring strategy (UUN + 3-day weighed dietary records) addresses this gap directly.

From a vascular assessment perspective, David et al. used pulse wave velocity (PWV) as their primary marker of vascular health—a measure of global arterial stiffness—without assessing endothelial function directly. Our study introduces flow-mediated dilation (FMD) of the brachial artery as the primary vascular endpoint, providing the first direct quantification of endothelium-dependent vasodilatory capacity (+1.7%, *p* < 0.01) in this clinical context, and establishing a mechanistic bridge between uremic toxin reduction and NO-mediated vascular remodeling underlying AVF maturation.

Crucially, David et al. did not measure uremic toxins, leaving the molecular mechanisms underlying their observed vascular benefits uncharacterized. Our study is the first to quantify indoxyl sulfate and p-cresyl sulfate by HPLC in ESRD patients undergoing AVF preparation, documenting reductions of 38% and 43% respectively and providing a direct mechanistic link between dietary intervention, endothelial toxin clearance, and endothelial functional recovery.

In terms of patient population, David et al. explicitly excluded diabetic patients—a major limitation they themselves acknowledged, given that diabetic nephropathy is the leading cause of ESRD worldwide. Our cohort reflects real-world clinical complexity: 40% of patients had diabetic nephropathy and 30% hypertensive nephrosclerosis, making our findings more broadly generalizable. Furthermore, all AVFs in our series were created as distal radiocephalic fistulas at the wrist—historically the most technically demanding AVF type with the highest maturation failure risk—whereas David et al. included a mixed access population (distal, middle-arm, and upper-arm fistulas, with ~22% upper-arm AVFs). Despite this more challenging operative context, our maturation rate of 95% and CVC dependency of 0% compare favourably with David et al.’s 89.3% maturation rate and 7.1% CVC rate in their KA-supplemented group.

Finally, David et al. [14], performed biochemical assessments only at a single pre-operative time point and could not formally evaluate nutritional status, PEW, or sarcopenia due to the retrospective design. Our study provides longitudinal biochemical tracking at four time points and includes formal EWGSOP2-based sarcopenia screening, confirming the complete safety of the dietary regimen on muscle mass and function.

In summary, the two studies are complementary rather than overlapping: David et al. established the proof of concept that KA-supplemented dietary therapy improves AVF outcomes through vascular stiffness reduction, while the present work provides the first mechanistic characterization of this effect at the endothelial level, introducing uremic toxin quantification, FMD assessment, and a more clinically representative and nutritionally rigorous framework.

Beyond IS and PCS, the VLPD+KA intervention likely exerts additional beneficial effects on endothelial function through its impact on asymmetric dimethylarginine (ADMA)—a potent endogenous inhibitor of eNOS. In CKD patients, elevated ADMA levels accumulate as a result of impaired renal clearance and reduced activity of dimethylarginine dimethylaminohydrolase (DDAH), the enzyme responsible for its degradation [15]. ADMA contributes to eNOS uncoupling, which, rather than producing NO, generates superoxide anions that further amplify oxidative stress and endothelial injury [16]. The LPD + KA regimen has been shown by Teplan et al. to significantly reduce ADMA levels in obese CKD patients through mechanisms involving BMI reduction and improved glycemic metabolism, which in turn restores DDAH activity [17]. Although the current study population did not specifically target obese patients, the general metabolic improvements conferred by VLPD+KA—including better glycemic control, reduced oxidative stress, and decreased uremic toxin load—may similarly attenuate ADMA-mediated eNOS uncoupling, contributing to the observed gains in FMD and vascular NO bioavailability.

The concept of vascular pre-conditioning emerges as a pivotal mechanistic framework for interpreting our results. By initiating VLPD+KA therapy prior to AVF creation, we may have primed the endothelium to withstand the hemodynamic stress associated with surgical vascular manipulation and the subsequent increase in blood flow demand required for fistula maturation. This pre-conditioning effect is mechanistically plausible: reduced circulating uremic toxins diminish endothelial oxidative stress, improved NO bioavailability facilitates shear-stress-mediated vasodilation and outward remodeling, and reduced systemic inflammation limits neointimal proliferative responses that are a key driver of fistula failure [18]. This concept parallels the recognized benefits of pharmacological pre-conditioning strategies in cardiovascular surgery, but it is achieved here entirely through nutritional means—a clinically attractive feature given the already substantial polypharmacy burden of CKD patients. The duration of pre-conditioning treatment, the optimal timing before AVF creation, and the minimum threshold of FMD improvement required to predict AVF success are important questions that future prospective studies should address.

A further dimension of the protective vascular mechanism involves the improvement of calcium–phosphate metabolism. Hyperphosphatemia, characteristic of advanced CKD, promotes vascular calcification through the transdifferentiation of VSMCs into an osteoblast-like phenotype, increases vascular stiffness, and induces oxidative stress via inhibition of inducible NOS (iNOS) and activation of protein kinase C [19]. The VLPD, by reducing dietary phosphate intake, directly lowers serum phosphate levels, thereby mitigating these deleterious pathways. Furthermore, hyperphosphatemia drives an increase in fibroblast growth factor 23 (FGF23) and a corresponding decrease in its co-receptor Klotho, both of which have been independently implicated in endothelial dysfunction and accelerated vascular aging in CKD [20]. The improvement in phosphate homeostasis induced by the VLPD+KA regimen may therefore reduce FGF23 levels and partially restore Klotho expression, adding another layer of vascular protection to the intervention’s effects.

Insulin resistance (IR) represents an additional mechanistic target of the VLPD+KA regimen. In CKD patients, chronic low-grade inflammation, metabolic acidosis, vitamin D deficiency, and uremic toxin accumulation collectively impair the PI3K/Akt insulin signaling pathway in endothelial cells, reducing eNOS activation and promoting a compensatory shift toward the MAPK/ET-1 vasoconstrictor pathway [21]. This dual impairment—reduced vasodilation and enhanced vasoconstriction—creates a hostile vascular environment. KA supplementation has been shown to improve insulin sensitivity in CKD patients, likely through reduction of uremic toxin-mediated inflammation and improvement of metabolic acidosis [17]. The restoration of insulin signaling in endothelial cells would be expected to increase eNOS-derived NO production, reduce ET-1 release, and shift the vascular milieu toward a vasodilatory and anti-thrombotic phenotype—effects entirely consistent with the FMD improvements observed in the present study.

It is instructive to compare the endothelial benefits achieved by VLPD+KA with those attributed to pharmacological interventions in CKD. Sodium–glucose cotransporter-2 inhibitors (SGLT-2i) and non-steroidal mineralocorticoid receptor antagonists (MRAs), such as finerenone, have both demonstrated significant endothelial protective effects in CKD through mechanisms including reduction of oxidative stress, restoration of NO bioavailability, and reduction of vascular inflammation [4,22]. A meta-analysis of 26 clinical studies concluded that, among antidiabetic drug classes, only SGLT-2 inhibitors significantly enhanced FMD [21]. The magnitude of the FMD improvement achieved in the present study with a purely nutritional intervention is noteworthy and suggests that the VLPD+KA regimen may achieve endothelial benefits comparable to those of pharmacological agents—without the risks of dose-adjustment in impaired renal function, drug interactions, or adverse effects such as hyperkalemia. The combination of VLPD+KA with these pharmacological strategies, which addresses endothelial dysfunction through complementary and partially non-overlapping mechanisms, deserves investigation in future randomized controlled trials.

The present findings also invite consideration of whether further nutritional adjuncts could amplify the vascular benefits achieved by VLPD+KA. Extra virgin olive oil (EVOO) rich in minor polar compounds (MPCs)—particularly hydroxytyrosol, oleocanthal, and oleuropein complex—has been shown by Marrone et al. to exert significant cardioprotective effects in CKD patients, including reductions in oxidative stress biomarkers, inflammatory cytokines (CRP, TNF-alpha, IL-6), atherogenic lipid indices, and carotid intima-media thickness [23]. Oleocanthal, the phenolic compound responsible for EVOO’s ibuprofen-like sensory properties, inhibits COX enzymes and may therefore counteract platelet aggregation and neointimal hyperplasia—processes directly relevant to AVF patency. Similarly, the plant-dominant low-protein diet (PLADO LPD), characterized by a predominance of plant-based protein sources, has been shown to reduce the generation of gut-derived uremic toxins (IS, PCS, TMAO), improve gut microbiota eubiosis, and enhance eNOS activity and NO bioavailability [24]. Integrating elements of the PLADO diet and EVOO supplementation into the VLPD+KA framework represents a promising nutritional strategy for maximizing endothelial protection in pre-dialysis CKD patients.

Adapted physical activity (APA) represents another non-pharmacological intervention with documented endothelial benefits in CKD that may complement the effects of the VLPD+KA regimen. Aerobic exercise normalizes plasma ET-1 levels, reduces ADMA concentrations, improves the redox state, and enhances NO bioavailability through multiple mechanisms, including facilitation of L-arginine transport, prevention of eNOS uncoupling, and reduction of NADPH oxidase-derived superoxide production [25]. Resistance training has additionally been shown to evoke direct NO release and improve inflammatory and redox profiles in CKD patients [25]. The practical implementation of APA in the pre-dialysis setting—where patients are often symptomatic and physically deconditioned—requires careful individualization and medical supervision, but the potential synergy between structured exercise and the VLPD+KA-induced vascular pre-conditioning effect merits formal evaluation in dedicated prospective trials.

A mechanistic thread that deserves further consideration is the relationship between VLPD+KA therapy and premature vascular aging—a well-established feature of the CKD milieu. IS accumulation has been shown to increase the generation of endothelial-derived extracellular vesicles carrying pro-inflammatory and pro-senescent microRNAs (miRs), which can propagate endothelial dysfunction and immune dysregulation throughout the vasculature [26]. Reduction in IS levels, as achieved in the present study, may therefore attenuate the release of these pathological extracellular vesicles and blunt the signaling cascades driving accelerated vascular senescence. This hypothesis aligns with the broader concept of inflammaging in CKD—the interplay between chronic inflammation and accelerated biological aging—and suggests that VLPD+KA therapy may exert anti-aging vascular effects beyond those expected from simple uremic toxin removal alone.

### 4.3. Clinical Implications and Future Directions

The clinical implications of these findings are profound. The ability to maximize AVF maturation rates and entirely avoid CVC use represents a major advance in pre-dialysis care. Early fistula failure and CVC dependency are strongly associated with an increased risk of bacteremia, central venous stenosis, hospitalization, and all-cause mortality. Implementing KA therapy in advanced CKD patients scheduled for AVF creation could become a new standard of care, serving as a dual-purpose intervention: mitigating the uremic milieu and pre-conditioning the vasculature for a successful surgical outcome. To formally address the mechanistic question of whether the observed benefits are attributable to the dietary protein restriction, the KA supplementation, or their synergistic combination, we are currently developing a prospective, controlled study protocol with a substantially increased sample size. This planned study will incorporate multiple comparative arms—including a standard low-protein diet without supplementation, a VLPD without KA, and a VLPD+KA group—enabling formal statistical dissociation of the dietary and supplementation effects on endothelial function, uremic toxin burden, AVF maturation outcomes, and nutritional status. This comparative design will provide the rigorous, evidence-based attribution that the current exploratory study was not powered to deliver.

### 4.4. Limitations

The present study has several limitations that should be acknowledged. The single-center, observational design with a relatively small sample size limits the generalizability of the findings. Additionally, no formal a priori sample size calculation was performed, as this study was designed as a hypothesis-generating observational cohort based on consecutive eligible patients referred during the study period; the sample of 20 patients therefore constitutes a convenience sample. Formal power calculations based on the effect sizes observed here—namely an FMD improvement of 1.7% and an IS reduction of 38%—will inform the design of our planned confirmatory randomized controlled trial. The absence of a control group receiving standard-of-care nutritional management (without VLPD+KA) precludes direct attribution of the observed vascular improvements solely to the nutritional intervention. Inter-individual variability in adherence to the dietary regimen, which was assessed by dietitian-supervised monitoring but not biochemically verified in all cases (e.g., through 24-h urinary urea nitrogen), represents a further limitation. Moreover, the measurement of FMD, although performed according to a standardized protocol, is subject to technical variability inherent to ultrasound-based techniques, including operator experience and biological confounders such as menstrual cycle, mental stress, and circadian variation [4]. Future multicenter randomized controlled trials with larger sample sizes, longer follow-up periods, and comprehensive vascular phenotyping—incorporating pulse wave velocity (PWV), carotid intima-media thickness, circulating ADMA, adiponectin, and miR biomarkers—will be essential to confirm and extend these promising results.

## 5. Conclusions

From a clinical management perspective, the present study supports the proactive integration of VLPD+KA nutritional therapy into the pre-dialysis care pathway not only for its established renoprotective and metabolic benefits but also as a deliberate strategy to optimize vascular health ahead of hemodialysis access surgery. The exceptional AVF maturation rate of 95% achieved in our cohort has substantial health–economic implications: CVCs are associated with higher rates of infection, thrombosis, and mortality compared with AVFs [27], and their avoidance translates directly into improved patient safety, quality of life, and long-term dialysis outcomes. Our data also reinforce the importance of multidisciplinary team management—nephrologist, dietitian, and vascular surgeon—in optimizing the pre-dialysis period. The concept of vascular pre-conditioning through nutritional means, as illustrated by the present study, represents an emerging and underexplored paradigm in dialysis access medicine that warrants further investigation. In conclusion, VLPD+KA supplementation reduces the uremic toxin burden, attenuates systemic inflammation, and restores endothelial function in advanced CKD patients, translating into superior arteriovenous fistula outcomes. These findings establish the vascular pre-conditioning potential of this intervention and provide a mechanistic rationale for incorporating nutritional optimization as a cornerstone of hemodialysis access preparation.

## Figures and Tables

**Table 1:** Comprehensive Biochemical Panel: Baseline and 3-Month Values.

Parameter	Baseline (Mean ± SD)	3 Months (Mean ± SD)	*p*-Value
Serum Creatinine (mg/dL)	7.2 ± 1.4	6.9 ± 1.3	n.s.
BUN (mg/dL)	82 ± 14	54 ± 11	<0.001
eGFR (mL/min/1.73 m^2^)	10.2 ± 2.3	10.8 ± 2.1	n.s.
Serum Albumin (g/dL)	3.9 ± 0.3	4.0 ± 0.3	n.s.
Bicarbonate (mEq/L)	19.2 ± 2.1	22.1 ± 1.8	<0.01
Phosphate (mg/dL)	5.8 ± 0.9	4.6 ± 0.7	<0.01
Calcium (mg/dL)	8.6 ± 0.5	8.9 ± 0.4	n.s.
PTH (pg/mL)	512 ± 140	423 ± 132	<0.05
Hemoglobin (g/dL)	10.4 ± 1.2	10.8 ± 1.1	n.s.
Total Cholesterol (mg/dL)	188 ± 32	178 ± 28	n.s.
LDL Cholesterol (mg/dL)	112 ± 24	104 ± 22	n.s.
Triglycerides (mg/dL)	182 ± 45	162 ± 35	<0.05

BUN, blood urea nitrogen; eGFR, estimated glomerular filtration rate; PTH, intact parathyroid hormone; LDL, low density lipoprotein; SD, standard deviation; n.s., not significant.

**Table 2:** Dietary Compliance and Ketoacid Analogue (KA) Supplementation Data.

Parameter	Value
Mean body weight (kg)	65 ± 8
Prescribed protein intake (g/kg IBW/day)	0.3–0.4
Actual protein intake at 3 months (g/kg/day)	0.35 ± 0.04
Total protein intake (g/day)	22.8 ± 3.1
Total energy intake (kcal/kg/day)	32 ± 2
Carbohydrate intake (% total energy)	60.6 ± 4.8
Fat intake (% total energy)	35.0 ± 3.5
Protein intake (% total energy)	4.4 ± 0.5
KA supplementation (tablets/day)	13 ± 1
24-h UUN at baseline (g/24 h)	6.2 ± 1.3
24-h UUN at 3 months (g/24 h)	2.9 ± 0.6

IBW, ideal body weight; KA, ketoacid analogues (Ketosteril^®^); UUN, 24-hour urinary urea nitrogen; SD, standard deviation. Values are expressed as mean ± SD.

**Table 3:** AVF Maturation Parameters at Pre-operative Assessment and Monthly Follow-up.

Parameter	Pre-Operative	1 Month	2 Months	3 Months
Cephalic vein diameter (mm)	2.8 ± 0.4	4.2 ± 0.6	5.4 ± 0.7	6.3 ± 0.8
AVF blood flow volume (mL/min)	—	420 ± 95	558 ± 88	648 ± 102
Resistance Index (RI)	0.78 ± 0.06	0.74 ± 0.05	0.71 ± 0.05	0.69 ± 0.05
Maturation rate, n (%)	—	12 (80%)	17 (85%)	19 (95%)

AVF, arteriovenous fistula; RI, resistance index; SD, standard deviation. Maturation defined as cephalic vein diameter ≥ 6 mm and AVF flow ≥ 500 mL/min (DOQI criteria). — indicates not applicable. Values are expressed as mean ± SD.

**Table 4:** Changes in Vascular, Inflammatory, and Nutritional Parameters from Baseline to 3 Months.

Parameter	Baseline (Mean ± SD or Median [IQR])	3 Months (Mean ± SD or Median [IQR])	Change	*p*-Value
**Vascular & Endothelial Function**				
Flow-Mediated Dilation (FMD) (%)	4.2 ± 1.1	5.9 ± 1.3	1.70%	<0.01
Resistance Index (RI)	0.78 ± 0.06	0.69 ± 0.05	−0.09	<0.01
**Uraemic Toxins**				
Indoxyl Sulfate (IS) (µmol/L)	31.4 ± 8.2	19.5 ± 6.3	−38.00%	<0.001
p-Cresyl Sulfate (PCS) (µmol/L)	22.7 ± 5.5	13.0 ± 4.8	−43.00%	<0.001
**Inflammatory Markers**				
C-Reactive Protein (CRP) (mg/L)	3.2 [2.5–4.0]	1.1 [0.8–1.6]	−65.60%	<0.01
Erythrocyte Sedimentation Rate (ESR) (mm/h)	36 ± 10	22 ± 8	−38.90%	<0.01
**Body Composition & Muscle Function (BIA)**				
Phase Angle (°)	4.5 ± 0.7	4.7 ± 0.7	+0.2°	0.04
Handgrip Strength (kg)—overall	28.5 ± 4.8	29.1 ± 4.8	+0.6 kg	n.s.
Males (n = 12)	33.2 ± 3.8	33.9 ± 3.7	+0.7 kg	n.s.
Females (n = 8)	21.4 ± 3.1	21.8 ± 3.0	+0.4 kg	n.s.
Skeletal Muscle Mass Index (SMMI) (kg/m^2^)	-	-	-	-
Males (n = 12)	8.2 ± 0.9	8.3 ± 0.8	+0.1 kg/m^2^	n.s.
Females (n = 8)	6.1 ± 0.7	6.2 ± 0.6	+0.1 kg/m^2^	n.s.
Body Mass Index (BMI) (kg/m^2^)	24.7 ± 2.6	24.5 ± 2.6	−0.2 kg/m^2^	n.s.

**Abbreviations:** FMD, flow-mediated dilation; RI, resistance index; IS, Indoxyl sulfate; PCS, p-cresyl sulfate; CRP, C-reactive protein; ESR, erythrocyte sedimentation rate; BMI, body mass index; BIA, bioelectrical impedance analysis; SD, standard deviation; IQR, interquartile range; n.s., not significant.

## Data Availability

The data presented in this study are available upon request from the corresponding author. The data are not publicly available due to privacy and ethical restrictions.
